# Preparation of ^99m^Tc-Clomiphene Citrate as a Novel Agent for Breast Cancer Imaging

**DOI:** 10.5402/2012/581281

**Published:** 2012-11-14

**Authors:** Ismail Taha Ibrahim, Mohamed Taha Elkolally, Ibrahim Yousof Abd Elgany, Ahmed Abd Albary, Mohamed Hodhod Elsayed

**Affiliations:** ^1^Labeled Compound Department, Hot Lab Center, Egyptian Atomic Energy Authority, Cairo 11371, Egypt; ^2^Faculty of Pharmacy, Cairo University, Cairo, Egypt

## Abstract

The aim of this work was to develop a novel ^99m^Tc-labelled derivative based on triphenylethylene for breast cancer imaging. ^99m^Tc-Clomiphene was obtained with a radiochemical yield of 94.4% by adding ^99m^Tc to 1.5 mg Clomiphene citrate in the presence of 10 **μ**g SnCl_2_ at pH 7. The optimization of the labeling yield of Clomiphene citrate, with ^99m^Tc, is described. The reaction parameters that affect the labeling yield were studied to optimize the labeling conditions. Radiochemical purity of the final product has been verified by means of paper chromatography and paper electrophoresis. Ehrlich Ascites Carcinoma (EAC) as a model of breast cancer cells was injected intraperitoneally (IP) to produce ascites and intramuscularly (IM) to produce solid tumor. Biodistribution study was carried out by the injecting solution of ^99m^Tc-Clomiphene in normal and tumor bearing mice. The uptake in ascites was over 12.5 % injected dose per gram tissue body weight, at 1hr after injection and above 12% in solid tumor. The T/NT value for ^99m^Tc-Clomiphene complex was found to be 5.5 ± 0.4 which was higher than that of the commercially available ^99m^Tc-MIBI. This data revealed the localization of tracer in tumor tissue with high percent sufficient to use ^99m^Tc-Clomiphene as a promising tool for the diagnosis of breast cancer.

## 1. Introduction 

 The diagnosis of breast cancer is based on physical examination supported by mammography and fine-needle aspiration cytology or core biopsy. The increasing use of MRI and especially ultrasonography has improved diagnostic accuracy but there is still a need for additional diagnostic methods. One of the diagnostic tools to confirm or exclude breast cancer is scintimammography. Various radiopharmaceuticals were used in detecting that breast cancer ^99m^Tc-sestamibi (MIBI) is the most popular agent [1, 2]. But ^99m^Tc-MIBI blood clearance is known to be very rapid, with a half-life of only a few minutes [3]. Clinical *in vivo* studies also have established significant correlations between ^99m^Tc-sestamibi efflux from tumors and P-glycoprotein (P-gp) expression in cancer patients [4, 5]. High levels of P-gp in tumor may indeed cause rapid washout of MIBI, decreasing the net cellular uptake of the tracer [6]. Tumor models have shown low tumor-to-muscle ratios (1 : 1) for all studied cations, including ^99m^Tc-MIBI [7]. Therefore, there is an urgent need for a radio pharmaceutical product which can be prepared in house with a simple and rapid procedure. In addition should accumulate in cancer cells at a high concentration to give high target-to-nontarget ratios to give more accuracy.

 Estrogen is a breast epithelial cell mitogen, and through interaction with other hormones and growth factors, it contributes to the activation of protooncogenes, such as c-myc, cyclin D1, and cyclin E [8]. These genes mediate G1-to-S phase transition during normal cell-cycle progression.

**Figure 1:**
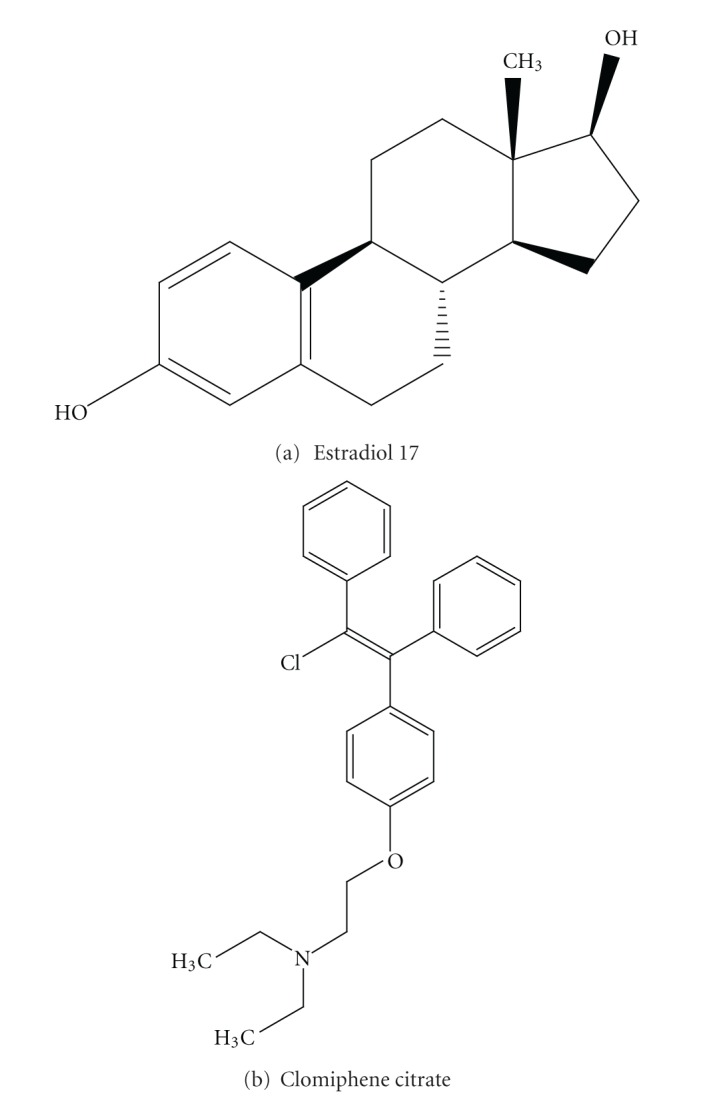
Chemical structure of E2 and Clomiphene citrate.

 Clomiphene citrate (CC) and related triarylethylene antiestrogens to antagonize the growth promoting effect of estrogens in target tissues have focused attention on the potential application of these compounds in estrogen receptor positive breast cancer treatment and prevention [9]. Clomiphene citrate is a triphenylethylene compound, belonging to a family of synthetic nonsteroidal estrogens/antiestrogens and selective estrogen receptor modulators (SERMs). These compounds, when compared to the natural estrogens, have both agonistic and antagonistic properties and mimic estrogen in shape, see (Figure 1) [10]. It binds with a high affinity to estrogen-receptor systems of target cells and to antiestrogen-specific binding sites that have been identified in the cytosols of estrogen-receptor positive tissues, for example, the mammary gland and uterus [11]. The constituent isomers of Clomiphene have been shown to suppress the proliferation of cultured human breast cancer cells [12] and to inhibit the growth of chemically induced breast cancer in the rat. It inhibits mammary gland development in rats [13]. Furthermore, Clomiphene showed antitumor activity in an early clinical trial for the treatment of advanced breast cancer [14]. *In vitro* studies have demonstrated that several Clomiphene citrate analogs have a direct antiproliferative activity in MCF-7 and LY2 breast tumor cell lines; *in vivo* studies showed Clomiphene citrate-related inhibition of MCF-7 breast tumor xenografts in nude mice [15]. Moreover Clomiphene is a potent inhibitor of PKC (protein kinase C) [16]. PKC inhibitors have been shown to decrease phosphorylation of P-glycoprotein [17, 18], drug-efflux activity [17] and P-glycoprotein drug binding [18]. 

 In this work Clomiphene was labeled with ^99m^Tc to be used as a potential agent for breast cancer imaging. Factors affecting the labeling yield will be studied in details.

## 2. Experimental

### 2.1. Materials

 Clomiphene citrate was a kind gift from Misr Pharmaceutical company, Cairo, Egypt. Stannous chloride dihydrate (SnCl_2_·52H_2_O, M.wt. = 225.6) was purchased from Sigma Chemical company, St. Louis, MO USA. Pertechnetate (^99m^TcO_4_^−^) was eluted from ^99^Mo/^99m^Tc generator, Elutec Brussels, Belgium. And all other chemicals were purchased from Merck and they were analytical reagents.

### 2.2. Animals

 Female Swiss albino mice weighing 20–25 g were purchased from the Institute of Eye Research Cairo, Egypt. The animals were kept at constant environmental and nutritional conditions throughout the experimental period and kept at room temperature (22 ± 2)°C with a 12 hr on/off light schedule. Female mice were used in this study due to their susceptibility to Ehrlich Ascites Carcinoma more than male mice [19]. Animals were kept with free access to food and water all over the experiment. The study was approved by the animal ethics committee, Labeled Compound Department, and was in accordance with the guidelines set out by the Egyptian Atomic Energy Authority.

### 2.3. Methods

**Figure 2:**
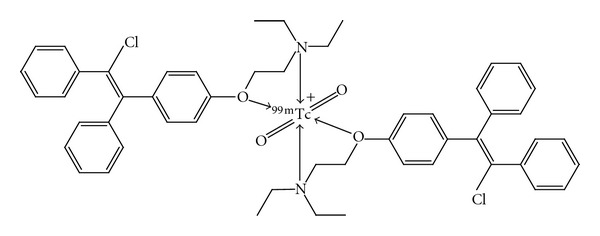
The chemical structure of ^99m^Tc-Clomiphene.

The reduced ^99m^Tc species are chemically reactive and combine with a wide variety of chelating compounds. Compounds containing atoms like O, N, S, and P usually can donate lone pair of electrons to form coordination bonds with ^99m^Tc (Figure 2). Accurately weighed 1.5 mg Clomiphene was transferred to a penicillin vial then the vial was evacuated. Exactly 10 *μ*m of SnCl_2_·2H_2_O solution was added and the pH 7 was adjusted. One milliliter of freshly eluted ^99m^TcO_4_ (400 MBq) was added to the above mixture. The reaction mixture was shaken by electric vortex and left at ambient temperature for a sufficient time to complete the reaction. For each labeling experiment, the radiochemical yield of the product was determined by paper chromatography and paper electrophoresis. The influence of various reaction parameters and conditions on radiolabelling efficiency, such as the amount of SnCl_2_·2H_2_O, concentration of substrate, pH of the reaction, reaction time, and reaction temperature, was investigated and optimized in order to maximize the radiochemical yield. Experiment studying each factor was repeated three times and differences in the data were evaluated with one-way ANOVA test. Results for *p* are reported and all the results are given as mean ± SEM. The level of significance was set at *p* = 0.05.

#### 2.3.1. Analysis of ^99m^Tc-Clomiphene Citrate

 Radiochemical yield and purity of the ^99m^Tc-Clomiphene Citrate were determined by paper chromatographic method using strips on two-paper sheet (1 cm width and 13 cm length); 1-2 *μ*L of the reaction mixture was placed 2 cm above the lower edge and was allowed to evaporate spontaneously, one strip was developed with acetone and other strip was developed with ethanol : water : ammonium hydroxide mixture (2 : 5 : 1). After complete development, the paper sheet was removed, dried, and cut into strips; each strip is 1 cm width, and then each strip was counted in a well-type c-counter. Radiochemical yield was further confirmed by paper electrophoresis. After filtration using 0.22 *μ*m Millipore filter to remove colloids and bacteria. On Whatman paper sheet (2 cm width and 47 cm length), 1-2 *μ*L of the reaction mixture was placed at 12 cm far from the cathode edge of the paper sheet. Electrophoresis is carried out for 1 h at voltage of 300 V using normal saline (0.9% w/v NaCl solution) as electrolytes source solution. After complete development, the paper was removed, dried, and cut into strips; each strip length was 1 cm, and then each strip was counted in a well-type c-counter. The percentage of radiochemical yield was calculated as the ratio of the radioactivity of ^99m^Tc-Clomiphene to the total activity multiplied by 100. While The percentage of stannous hydroxide colloid was determined by the filtration of the reaction mixture through 0.22 *μ*m Millipore filter [20] using a suitable pressure and according to

(1) %colloid  =Activity  before  filtration−Activity  after  filtrationActivity  before  filtration   ×100.

#### 2.3.2. Determination of *In Vitro* Stability of ^99m^Tc-Clomiphene

 The reaction mixture was left at ambient temperature for 48 h and 1-2 *μ*L samples were taken from it at different time intervals. The radiochemical yield of the samples was measured by paper chromatography and paper electrophoresis.

#### 2.3.3. Determination of the Partition Coefficient of ^99m^Tc-Clomiphene

The partition coefficient was determined by mixing 50 *μ*L of ^99m^Tc-Clomiphene solution with equal volumes of 1-octanol and phosphate buffer (0.025 M at pH 7.4) in a centrifuge tube. The mixture was vortexed at room temperature for1 min and then centrifuged at 5,000 rpm for 5 min. Subsequently 100 *μ*L samples from the 1-octanol and aqueous layers were pipetted into other test tubes and counted in a gamma counter. The measurement was repeated three times. The partition coefficient value was expressed as log⁡⁡*p* [21]

(2) $$\begin{array}{c} \log p_{\text{oct}/\text{wat}}=\left(\frac{{\left[\text{solute}\right]}_{\text{octanol}}}{{\left[\text{solute}\right]}_{\text{deionized}\;\text{water}}}\right). \end{array}$$

#### 2.3.4. Induction of Tumor in Mice

 Ehrlich tumor is an experimental model for breast cancer. Ehrlich ascites tumor had been derived from a murine mammary carcinoma [22].

The use of Ehrlich Ascites Carcinoma (EAC) as a model in anticancer research was proven by many authors to give accurate and reliable results [23]. EAC was maintained in female Swiss albino mice through weekly IP transplantation of 2.5 × 10^6^ tumor cells/mouse. EAC cells were obtained by needle aspiration with aseptic condition. The ascitic fluid was diluted with sterile saline so that 0.1 mL contains 2.5 × 10^6^ cells counted microscopically using a haemocytometer. 0.2 mL solution was then injected intraperitoneally to produce ascites and intramuscularly in the right thigh to produce solid tumor where the left is kept as control. The animals were maintained for 10–15 days till the tumor development.

#### 2.3.5. Biodistribution Studies of the ^99m^Tc-Clomiphene

 The experimental procedures of the biological studies were done in accordance with the guidelines set out by the Egyptian Atomic Energy Authority and were approved by the animal ethics committee, Labeled Compound Department. Biodistribution studies of the ^99m^Tc-Clomiphene in normal and tumor bearing Swiss albino mice of body mass 20–25 g (*n* = 6) were carried out at 15, 30, and 60 min and 4, 24 hr after injection. Prior to the study, animals were housed in groups of six and provided with food and water. Aliquots of 10 *μ*L ^99m^Tc-Clomiphene were injected into each mouce via the tail vein. Each mouce was weighed then anaesthetized by chloroform. Samples of fresh blood bone and muscle were collected in preweighed vials and counted. Blood, bone, and muscles were assumed to be 7, 10, and 40% of the total body weight, respectively. Organs and tissues were rinsed with saline, collected in plastic containers, and weighed. The radioactivity of each sample as well as the back ground was counted in a well-type NaI (Tl) crystal coupled to SR-7 scalar ratemeter. Percentage of injected dose per gram (% ID/g ± SD) in a population of six mice for each time point is reported. Data were evaluated with one-way ANOVA test. Results for *p* are reported and all the results are given as mean ± SEM. The level of significance was set at *p* = 0.05.

## 3. Result and Discussion

**Figure 3:**
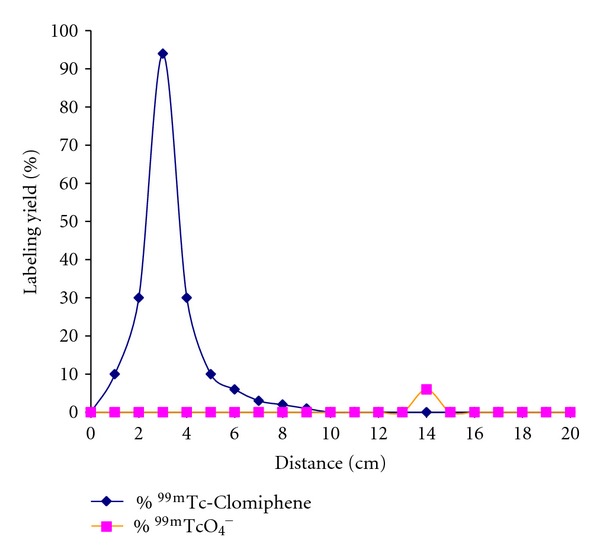
Electrophoresis radiochromatogram of ^99m^Tc-Clomiphene.

The Radiochemical purity of ^99m^Tc-Clomiphene was determined using paper chromatography where free ^99m^Tc remained near the origin (RF = 0-0.1), while ^99m^Tc-Clomiphene moved with the solvent front (RF = 0.8). Radiochemical purity was further confirmed by paper electrophoresis.  Figure 3 can determine the charge character of the prepared ^99m^Tc-Clomiphene complex, where the unreacted ^99m^TcO_4_^−^) or anionic species and ^99m^Tc-Clomiphene moved to different distances away from the spotting point towards the anode depending on the charge of each one (distance from spotting point = 14 and 3 cm, resp.). This can be explained because of the positive charge of ^99m^Tc-Clomiphene complex. Results of radiochemical yield from the two separation methods (paper chromatography and paper electrophoresis) are nearly the same.

### 3.1. Factors Affecting the Labeling Yield

#### 3.1.1. Effect of Reaction Time

**Figure 4:**
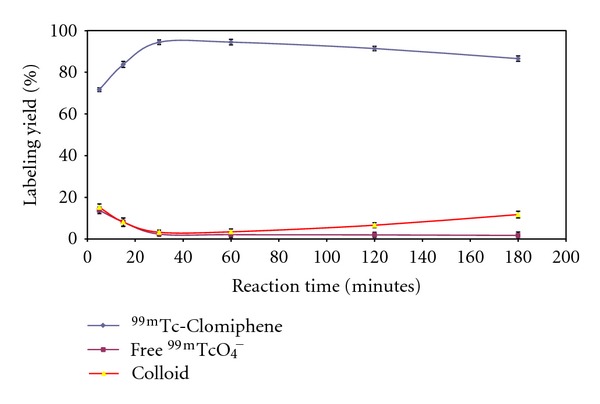
^99m^Tc-Clomiphene yields versus reaction time; 1.5 mg Clomiphene, 10 *μ*g of SnCl_2_·2H_2_O, and 1 mL (*400 MBq) of ^99m^TCO_4_ at pH 7; the reaction mixture was kept at room temperature at different times after labeling.

The radiochemical yield of ^99m^Tc-Clomiphene was studied at different reaction times (5–180 min).  Figure 4 shows that the rate of the formation of ^99m^Tc-Clomiphene was started relatively slowly with a yield of 71.7 ± 0.92%, at 5 min. The highest yield of 94.4 ± 1.1% was achieved at 30 min reaction time. The radiochemical yield reaches the saturation value and is not affected by increasing the reaction time above 30 min.

#### 3.1.2. Effect of Clomiphene Amount

**Figure 5:**
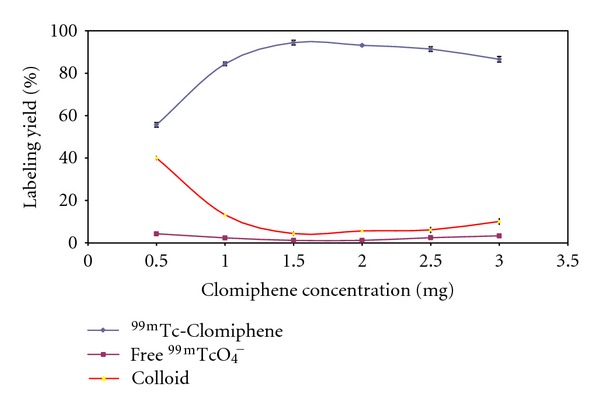
Percent labeling yield of Clomiphene with ^99m^TC as a function of substrate concentration; reaction conditions: 0.5–3 mg Clomiphene, 10 *μ*g of SnCl_2_·2H_2_O, and 1 mL (*400 MBq) of ^99m^TcO_4_^−^ at pH 7; the reaction mixture was kept at room temperature for 30 min.

As shown in Figure 5. At low Clomiphene concentration (0.5 mg) the yield was small and equal to 62.2 ± 1.1%. This low labeling yield was due to the Clomiphene concentration being insufficient to form complex with all of the reduced technetium so, the remaining reduced technetium-99 m was converted to reduce hydrolyzed technetium colloid (34.2 ± 0.97%). Increasing the substrate concentration leads to higher labeling yield and the maximum yield of 94.4 ± 1% was achieved at 1.5 mg clomiphene. By increasing Clomiphene concentration over the optimum value, the labeling yield was decreased.

#### 3.1.3. Effect of SnCl_2_·2H_2_O Concentration

**Figure 6:**
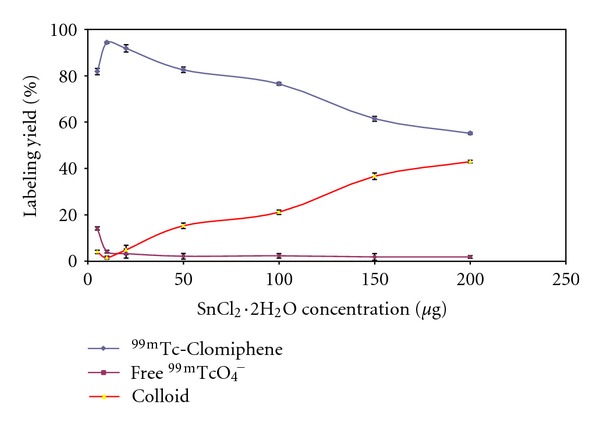
Effect of SnCl_2_·2H_2_O concentration on the labeling yield of ^99m^Tc-clomiphene; reaction conditions: 1.5 mg clomiphene, 5–200 *μ*g of SnCl_2_·2H_2_O, and 1 mL (*400 MBq) of ^99m^TcO_4_^−^ at pH 7; the reaction mixture was kept at room temperature for 30 min.

As shown in Figure 6, the radiochemical yield was dependent on the amount of SnCl_2_·2H_2_O present in the reaction mixture. At 5 *μ*g SnCl_2_·2H_2_O, the labeling yield of ^99m^Tc-Clomiphene was 81.8 ± 0.35% due to the fact that SnCl_2_·2H_2_O concentration was insufficient to reduce all pertechnetate so the percentage of ^99m^TcO_4_^−^ was relatively high (14.13 ± 0.86%). The labeling yield significantly increased by increasing the amount of SnCl_2_·2H_2_O from 5 to 10 *μ*g (optimum content), at which a maximum labeling yield of 94.4 ± 0.35% was obtained. By increasing the amount of SnCl_2_·2H_2_O above the optimum concentration value, the labeling yield decreased again because the excess SnCl_2_·2H_2_O was converted to colloid (36.67 ± 1.42% at 150 *μ*g SnCl_2_·2H_2_O).

#### 3.1.4. Effect of pH of the Reaction Mixture

**Figure 7:**
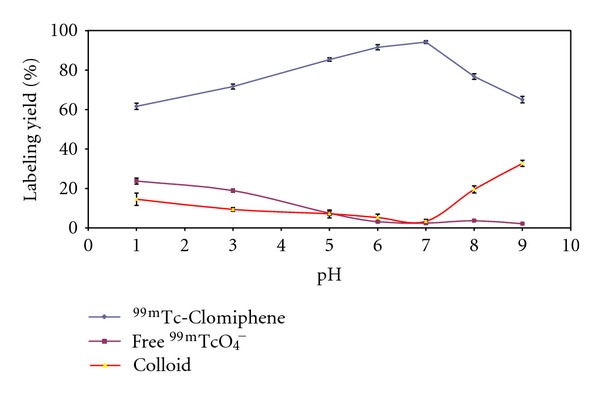
Effect of pH on the labeling yield of Clomiphene with ^99m^Tc; 1.5 mg Clomiphene, 10 *μ*g of SnCl_2_·2H_2_O, and 1 mL (*400 MBq) of ^99m^TcO_4_^−^ at 1–9 pH; the reaction mixture was kept at room temperature for 30 min.

As shown in Figure 7, at pH 1 the labeling yield of ^99m^Tc-Clomiphene complex was small and equal to 61.8 ± 1.6% and this yield increased with increasing the pH of the reaction mixture where pH 7 gave the maximum labeling yield of 94.4 ± 0.7%. By increasing the pH greater than 7, the labeling yield decreased again till it became 65.03 ± 1.65% at pH 9, where colloid was the main impurity (32.7 ± 1.57% at pH 9).

#### 3.1.5. Effect of Reaction Temperature (Figure 8)

***In Vitro* Stability of ^99m^Tc-Clomiphene**

**Figure 9:**
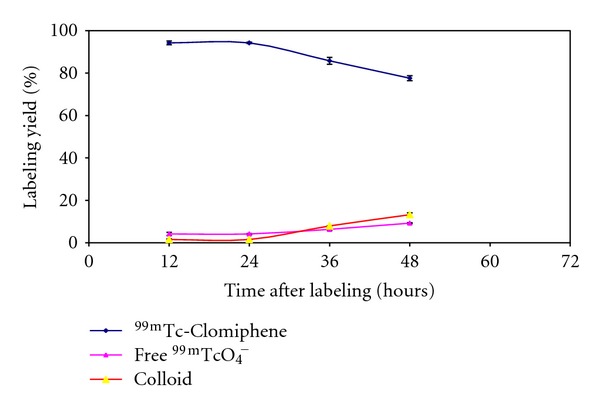
*In vitro* stability of ^99m^Tc-Clomiphene.

*In vitro* stability of ^99m^Tc-Clomiphene was studied in order to determine the suitable time for injection to avoid the formation of the undesired products that result from the radiolysis of the labeled compound. These undesired radioactive products might be accumulated in nontarget organs. The results of stability showed that the ^99m^Tc-Clomiphene is stable up to 24 h as shown in Figure 9.

**Partition Coefficient of ^99m^Tc-Clomiphene**

Increased lipophilicity within a certain range facilitates the inward transport of positively charged ions through the membrane lipid bilayer, as has been shown for several lipophilic cations, including technetium complexes and phosphonium cations. log⁡⁡*p* of ^99m^Tc-Clomiphene equals 3.67 ± 0.01, showing that ^99m^Tc-Clomiphene is lipophilic compound and ^99m^Tc-MIBI has a lower lipophilic nature (log⁡⁡*p*1.2) [24] than it. As a result it fulfills the requirement which is essential for high retention in cancer cells.

#### 3.1.6. Biodistribution of ^99m^Tc-Clomophene

- Biodistribution of ^99m^Tc-Clomiphene in normal Swiss albino mice (Table 1),
- biodistribution of ^99m^Tc-Clomiphene in ascites bearing mice (Table 2),
- biodistribution in solid tumor bearing mice (Table 3).

## 4. Conclusion

Clomiphene was labeled with ^99m^Tc with a high log⁡⁡*p* value and labeling yield of 94.4% using SnCl_2_·2H_2_O as a reducing agent. This study showed good *in vitro* and *in vivo* stability of the ^99m^Tc-Clomiphene. Upon the injection of ^99m^Tc-Clomiphene in both normal and tumor bearing mice, it was found that the high uptake of radioactivity in kidneys indicates that excretion of ^99m^Tc-Clomiphene occurs mainly through the urinary system. Cancer cells uptake of ^99m^Tc-Clomiphene is 12.6 % ID/g at 1 h after injection. ^99m^Tc-Clomiphene accumulation was 5.5 times greater in solid tumor than in normal muscle tissue. It showed high retention in cancer cells. The great incorporation of ^99m^Tc-Clomiphene in tumor sites (ascites or solid tumor) facilitates tumor imaging. ^99m^Tc-Clomiphene is not affected by P-gp, so it overcomes the limitations of ^99m^Tc-MIBI. As a result, ^99m^Tc-Clomiphene is a novel radiopharmaceutical for breast cancer Imaging and safeer than the commercially available ^99m^Tc-MIBI. In conclusion, this study demonstrates a hopeful approach for cancer imaging.

## Figures and Tables

**Figure 8:**
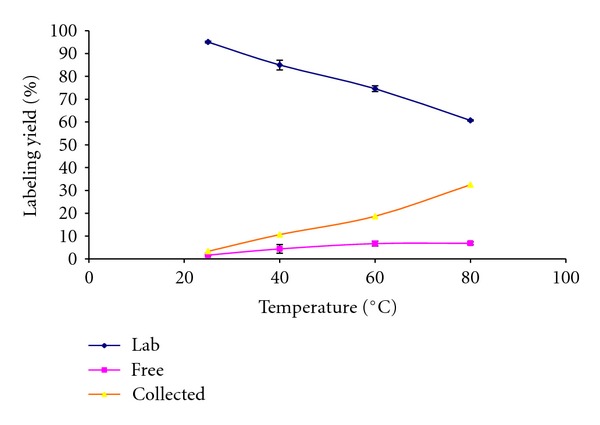
Variation of the radiochemical yield of ^99m^Tc-Clomiphene as a function of reaction temperature.

**Table 1:** Biodistribution of ^99m^Tc-Clomiphene in normal Swiss albino mice at different time intervals after injection.

Organs and body fluids	Percent ID/organ				
	Time after injection				
	15 min	30 min	1 hr	4 hr	24 hr
Blood	17.8 ± 1.8	18.0 ± 1.2	12.7 ± 1.1	6.5 ± 0.4	3.6 ± 0.2
Kidneys	18.3 ± 0.3	17.7 ± 1.1	15.5 ± 0.7	11.1 ± 1.1	3.1 ± 0.5
Liver	4.2 ± 0.3	3.6 ± 0.1	3.0 ± 0.2	2.1 ± 0.4	0.9 ± 0.2
Spleen	2.0 ± 0.2	3.2 ± 0.4	2.9 ± 0.7	2.3 ± 0.4	1.8 ± 0.7
Intestine	4.9 ± 1.2	5.2 ± 1.4	6.4 ± 1.5	6.4 ± 0.9	7.4 ± 0.5
Stomach	7.1 ± 0.6	10.3 ± 1.3	13.1 ± 1.0	11.7 ± 1.2	8.3 ± 0.5
Lungs	5.5 ± 0.1	3.3 ± 0.7	2.9 ± 0.5	2.4 ± 0.4	1.1 ± 0.4
Heart	12.0 ± 0.6	7.0 ± 0.7	3.5 ± 0.6	3.4 ± 0.4	1.5 ± 0.1
Thyroid	0.3 ± 0.03	0.9 ± 0.2	0.9 ± 0.3	1.4 ± 0.2	0.7 ± 0.3
Muscle	0.9 ± 0.2	2.0 ± 0.5	2.7 ± 0.7	3.3 ± 0.3	2.6 ± 0.1
Bone	0.6 ± 0.1	0.7 ± 0.3	1.2 ± 0.1	1.4 ± 0.3	2.0 ± 0.4

ID/gram ± S.D, *n* = 6.

**Table 2:** Biodistribution of ^99m^Tc-Clomiphene in Swiss albino ascites bearing mice at different time intervals after injection.

Organs and body fluids	Percent ID/organ				
	Time after injection				
	15 min	30 min	1 hr	4 hr	24 hr
Blood	16.5 ± 1.1	13.2 ± 0.6	8.4 ± 1.3	4.3 ± 1.3	1.5 ± 0.0
Kidneys	19.1 ± 0.6	16.2 ± 2.5	15.4 ± 0.7	12.2 ± 1.2	3.2 ± 0.0
Liver	3.7 ± 0.3	3.1 ± 0.6	2.2 ± 0.6	2.0 ± 0.5	1.8 ± 0.7
Spleen	2.5 ± 0.1	2.7 ± 0.2	2.9 ± 0.1	2.9 ± 0.5	2.7 ± 0.3
Intestine	4.6 ± 0.5	5.3 ± 0.6	6.7 ± 0.4	7.3 ± 1.1	7.8 ± 0.5
Stomach	6.2 ± 0.3	8.4 ± 1.8	9.3 ± 0.8	11.6 ± 1.2	7.8 ± 0.5
Lungs	7.5 ± 0.1	4.3 ± 0.5	2.6 ± 0.2	1.9 ± 0.3	1.4 ± 0.2
Heart	8.0 ± 0.3	7.1 ± 1.2	2.2 ± 0.2	2.1 ± 0.9	1.5 ± 0.1
Thyroid	0.5 ± 0.03	1.0 ± 0.5	1.4 ± 0.02	1.9 ± 0.6	1.2 ± 0.1
Muscle	1.3 ± 0.1	1.9 ± 0.4	2.6 ± 0.2	3.9 ± 1.1	2.6 ± 0.3
Bone	1.0 ± 0.1	1.2 ± 0.1	3.0 ± 0.1	2.2 ± 0.7	2.3 ± 0.3
Ascites	3.30 ± 0.3	5.4 ± 1.2	12.6 ± 0.4	7.8 ± 0.8	4.6 ± 0.2

% ID/gram ± S.D, *n* = 6.

**Table 3:** Biodistribution of ^99m^Tc-Clomiphene in Swiss albino solid tumor bearing mice at different time intervals after injection.

Organs and body fluids	Percent ID/organ				
	Time after injection				
	15 min	30 min	1 hr	4 hr	24 hr
Blood	16.8 ± 1.8	13.8 ± 0.9	6.4 ± 1.1	3.2 ± 0.5	2.3 ± 0.7
Kidneys	18.9 ± 0.9	18.2 ± 0.9	13.8 ± 1.3	9.1 ± 1.3	3.8 ± 0.7
Liver	4.4 ± 0.27	3.2 ± 0.1	3.5 ± 0.1	3.3 ± 0.4	2.1 ± 0.1
Spleen	1.7 ± 0.1	1.8 ± 0.4	2.1 ± 0.7	1.6 ± 0.25	1.2 ± 0.4
Intestine	5.2 ± 0.2	5.2 ± 1.3	6.9 ± 1.2	7.2 ± 0.9	7.5 ± 0.5
Stomach	8.1 ± 0.6	9.8 ± 1.2	11.8 ± 0.5	12.3 ± 1.1	5.5 ± 1.2
Lungs	5.5 ± 0.1	3.3 ± 0.5	1.9 ± 0.4	1.7 ± 0.7	1.9 ± 0.6
Heart	7.0 ± 0.8	4.5 ± 1.5	2.5 ± 0.7	2.2 ± 0.7	1.1 ± 0.4
Thyroid	0.8 ± 0.02	1.2 ± 0.4	1.3 ± 0.7	1.7 ± 0.23	0.6 ± 0.3
Normal muscle	0.8 ± 0.05	2.9 ± 0.6	2.3 ± 0.6	3.1 ± 0.9	2.9 ± 0.5
Bone	1.2 ± 0.1	1.6 ± 0.5	1.4 ± 0.7	1.2 ± 0.4	0.7 ± 0.5
Tumor muscle	2.7 ± 0.2	5.6 ± 0.9	12.5 ± 1.8	8.8 ± 1.1	4.2 ± 0.9

% ID/gram ± S.D, *n* = 6.

## References

[1] Khalkhali I, Cutrone JA, Mena IG (1995). Scintimammography: the complementary role of Tc-99m sestamibi prone breast imaging for the diagnosis of breast carcinoma. *Radiology*.

[2] Palmedo H, Grünwald F, Bender H (1996). Scintimammography with technetium-99m methoxyisobutylisonitrile: comparison with mammography and magnetic resonance imaging. *European Journal of Nuclear Medicine*.

[3] Wackers FJT, Berman DS, Maddahi J (1989). Technetium-99m hexakis 2-methoxyisobutyl isonitrile: human biodistribution, dosimetry, safety, and preliminary comparison to thallium-201 for myocardial perfusion imaging. *Journal of Nuclear Medicine*.

[4] Sun SS, Hsieh JF, Tsai SC, Ho YJ, Lee JK, Kao CH (2000). Expression of mediated P-glycoprotein multidrug resistance related to Tc-99m MIBI scintimammography results. *Cancer Letters*.

[5] Filipits M, Suchomel RW, Dekan G (1996). MRP and MDR1 gene expression in primary breast carcinomas. *Clinical Cancer Research*.

[6] Del Vecchio S, Ciarmiello A, Potena MI (1997). In vivo detection of multidrug-resistant (MDR1) phenotype by technetium-99m sestamibi scan in untreated breast cancer patients. *European Journal of Nuclear Medicine*.

[7] Barbarics E, Kronauge JF, Davison A, Jones AG (1998). Uptake of cationic technetium complexes in cultured human carcinoma cells and human xenografts. *Nuclear Medicine and Biology*.

[8] Butt AJ, Caldon CE, McNeil CM, Swarbrick A, Musgrove EA, Sutherland RL (2008). Cell cycle machinery: links with genesis and treatment of breast cancer. *Advances in Experimental Medicine and Biology*.

[9] Cuzick J, Wang DY, Bulbrook RD (1986). The prevention of breast cancer. *The Lancet*.

[10] Shelly W, Draper MW, Krishnan V, Wong M, Jaffe RB (2008). Selective estrogen receptor modulators: an update on recent clinical findings. *Obstetrical and Gynecological Survey*.

[11] Gazit A, Livshitz T, Shani J (1987). Fluoro-clomiphene and its synthetic precursors: synthesis and receptor binding. *Steroids*.

[12] Murphy LC, Sutherland RL (1983). Antitumor activity of clomiphene analogs in vitro: relationship to affinity for the estrogen receptor and another high affinity antiestrogen-binding site. *Journal of Clinical Endocrinology and Metabolism*.

[13] Richards JF, Griffith DR (1974). Effect of cis and trans clomiphene on mammary gland development in the rat. *Fertility and Sterility*.

[14] Hecker E, Vegh I, Levy CM (1974). Clinical trial of clomiphene in advanced breast cancer. *European Journal of Cancer and Clinical Oncology*.

[15] Baumann RJ, Bush TL, Cross-Doersen DE (1998). Clomiphene analogs with activity in vitro and in vivo against human breast cancer cells. *Biochemical Pharmacology*.

[16] O’Brian CA, Liskamp RM, Solomon DH, Weinstein IB (1986). Triphenylethylenes: a new class of protein kinase C inhibitors. *Journal of the National Cancer Institute*.

[17] Chambers TC, McAvoy EM, Jacobs JW, Eilon G (1990). Protein kinase C phosphorylates P-glycoprotein in multidrug resistant human KB carcinoma cells. *The Journal of Biological Chemistry*.

[18] Bates SE, Lee JS, Dickstein B, Spolyar M, Fojo AT (1993). Differential modulation of P-glycoprotein transport by protein kinase inhibition. *Biochemistry*.

[19] Abu-Zeid M, Hori H, Nagasawa H, Uto Y, Inayama S (2000). Studies of methyl 2-nitroimidazole-1-acetohydroxamate (KIN-804) 2: effect on certain antioxidant enzyme systems in mice bearing Ehrlich ascites carcinoma. *Biological and Pharmaceutical Bulletin*.

[20] Celerier C, Robert N, Bertrand-Barat J, Ducassou D (1997). Nuclear medicine functional imaging. *Médecine Nucléaire Imagerie Fonctionnelle et Métabolique*.

[21] Liu FEI, Youfeng HE, Luo Z

[22] Lettré R, Paweletz N, Werner D, Granzow C (1972). Sublines of the ehrlich-lettré mouse ascites tumour a new tool for experimental cell research. *Die Naturwissenschaften*.

[23] Sheeja KR, Kuttan G, Kuttan R (1997). Cytotoxic and antitumour activity of Berberin. *Amala Research Bulletin*.

[24] Lampidis TJ, Shi YF, Calderon CL, Kolonias D, Tapiero H, Savaraj N (1997). Accumulation of simple organic cations correlates with differential cytotoxicity in multidrug-resistant and -sensitive human and rodent cells. *Leukemia*.

